# A comparative evaluation: Oral leukoplakia surgical management using diode laser, CO2 laser, and cryosurgery

**DOI:** 10.4317/jced.53602

**Published:** 2017-06-01

**Authors:** Madhukar Natekar, Hosahallli-Puttaiah Raghuveer, Dilip-Kumar Rayapati, Eshwara-Singh Shobha, Nagesh-Tavane Prashanth, Vinod Rangan, Archana G Panicker

**Affiliations:** 1 Post Graduate Student , Department of Oral and Maxillofacial Surgery, Dayananda Sagar College of Dental Sciences; 2 MDS, Principal and Head of Department of Oral and Maxillofacial Surgery, Dayananda Sagar College of Dental Sciences; 3 MDS,Professor, Department of Oral and Maxillofacial Surgery, Dayananda Sagar College of Dental Sciences; 4 MDS, Associate Professor, Department of Oral and Maxillofacial Surgery, Dayananda Sagar College of Dental Sciences; 5 MDS, Assistant Professor, Department of Oral and Maxillofacial Surgery, Dayananda Sagar College of Dental Sciences

## Abstract

**Background:**

The comparatively evaluate the three surgical treatment modalities namely cryosurgery, diode and CO2 laser surgery in terms of healing outcomes on the day of surgery, first and second week post operatively and recurrence at the end of 18 months was assessed.

**Material and Methods:**

Thirty selected patients were divided randomly into three groups. Each group comprising of ten patients were subjected to one of the three modalities of treatment namely cryosurgery, diode laser or CO2 laser surgery for ablation of OL. Obtained data was analyzed using mainly using Chi-square and Anova tests.

**Results:**

Study showed statistical significant differences (p > 0.05) for evaluation parameters like pain, edema and scar. The parameters like infection, recurrence, bleeding showed no statistical significance. Pain was significantly higher in CO2 laser surgery group as compared with diode laser group. There was no recurrence observed at the end of the 6 months follow up period in all the three study groups.

**Conclusions:**

Observations from the study highlights that all three surgical modalities used in this study were effective for treatment of OL, and the overall summation of the results of the study showed that laser therapy (CO2 and Diode) seems to offer better clinically significant results than cryotherapy.

** Key words:**Oral premalignant lesion, leukoplakia, cryosurgery, CO2 laser surgery, diode laser surgery.

## Introduction

Oral cancer is one of the commonest of all cancers in India and only 10% are non-tobacco related (1). Oral leukoplakia (OL), a commonly found oral lesion, precedes oral cancer development in certain section of people (2). This precancerous white lesion poses a significant risk of malignant transformation and the rate ranges from 0.13 % to 34% (3). It is one among the common premalignant lesions of the oral mucosa and tobacco is the usual notable etiological factor (4). The risk of malignant transforma-tion of OL is difficult to assess as suggested by Napier and Speight who reviewed clinical factors like age, sex, location and type of lesion responsible for the malignant transformation of OL and found that the result varied in different studies (5). It is a premalignant lesion usually associated with tobacco usage and other factors being betel nut chewing and alcohol consumption (6).

The first line in the management of OL is elimination of contributory factors like smoking, tobacco usage , quitting betel quid, and reduction or complete withdrawal of alcohol usage. Complete cessation of tobacco can have substantial implication on prognosis (7). The line of treatment is also based on the lesion severity including its site, size, and location with any associated dysplasia (8).

OL is managed by various modalities of treatment both medical and surgical. The goal of OL is to reverse or totally eradicate the changes which has happened in the oral mucosa. OL is treated through the medical line of management with varied success, both topically and systemically, and it includes agents like vitamin A and retinoid, systemic beta-carotene, lycopene, ketorolac local bleomycin (9). Although vitamin A or retinoid, have been used as a medical line of management little scientific evidence exists for their use with regard to reducing rate of recurrence and its malignant transformation rates (10).

Various surgical treatment modalities for OL management have been advocated and it includes conventional surgery, cryotherapy and laser surgery (8,11). Conventional surgeries are limited by site and extent of the lesion. Cryosurgery is simple, safe, time tested and an effective modality of treatment for OL and also for treating certain benign and pre-cancer oral cavity lesions. Lasers provide a bundle of options over conventional surgery as they provide advantage of precision excision, homeostasis, reduced postoperative swelling and pain (12,13). This study was designed to investigate the efficacy and compare the sequel of healing and recurrence pattern of three surgical modalities – Cryosurgery, CO2 laser, and Diode Laser surgery in treating OL.

## Material and Methods

Thirty outpatients visiting the Department of Oral and Maxillofacial Surgery of Dayananda Sagar College of Dental Sciences, Bangalore, who presented with persistent white lesion six weeks’ status post habit cessation and with a biopsy result positive for OL were enrolled for the study. An informed written consent was secured from all patients and advantages and disadvantages of surgery were explained. Patients were subjected to blood investigations including complete hemogram, random blood sugar, bleeding time, clotting time. This clinical study was endorsed by the ethical panel or the institutional review board. Those patients with OL that regressed in size following habit cessation and those patients with active malignancy were excluded from the study. The study group comprised of 20 men and 10 women with a mean age of 36 years.

Total of three groups was formed by dividing thirty patients randomly with each group consisting of ten patients. Each group was subjected to one of the three different treatment modes. Group, I patients were treated using a diode laser, Group II patients were treated using CO2 laser and Group III was treated using nitrous oxide cryosurgery. All the patients were evaluated using a questionnaire to obtain past medical, surgical, and dental history and Intra and extra oral examination was performed. Two percent Lignocaine with epinephrine 1:80,000 was infiltrated into the surgical area as local anesthetic agent for all groups.

**Figure 1:**
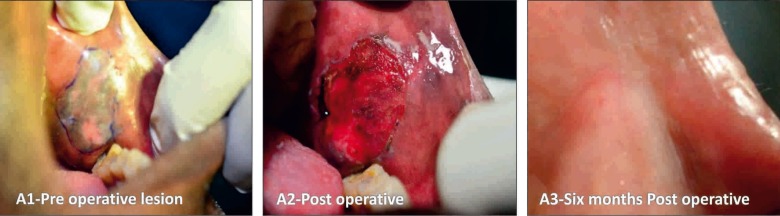
Diode laser surgery. A1) Pre operative lesion. A2) Post operative. A3) 6 months Post operative.

**Figure 2:**
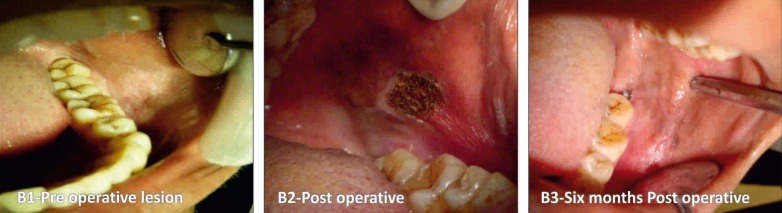
CO2 laser varorization. B1) Pre operative lesion. B2) Post operative. B3) 6 months Post operative.

**Figure 3:**
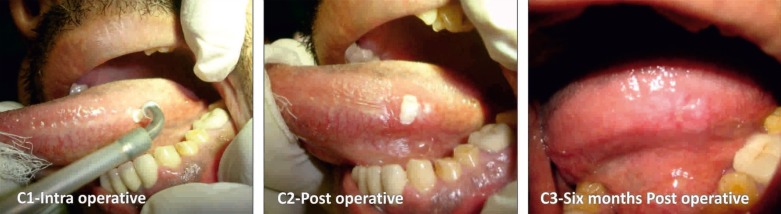
Cryosurgery. C1) Intra operative. C2) Post operative. C3) 6 months Post operative.

In Group I: A 970 nm diode laser system with 4 watts was used in a continuous mode under local anesthesia. The leukoplakic lesions were identified and limits of the lesion were marked using a surgical marker. The treatment was performed in contact mode until lesion was completely ablated (Fig. 1). In Group II: A CO2 laser was used to perform surgery by a wavelength beam of 10.6 μm. The power output was maintained in the usual standard range of 5 - 15 watt on pulsed/continuous mode, while the aiming beam with a spot size of 1mm (standard) was used. The tissue to be ablated was initially marked with a margin of about 3mm in a pulsed mode and the ablation was performed in a non-contact mode by moving a slightly defocused spot of CO2 laser on the lesion until complete evaporation and it reached sub-mucosa (Fig. 2). In Group III: The cryosurgery procedure was performed by direct application of nitrous oxide cryoprobe at a temperature of -65◦ to -85◦ C. The cryoprobe was applied for around 45-60 seconds following ice ball formation and the tissue was allowed to thaw completely prior to reapplication of the probe. The cryoprobe was applied for at least two or three freeze-thaw cycles. The probe was successively applied over adjacent sites and a spatial overlap consisting of rapid frozen areas were produced. Several applications by mapping technique were necessary to treat large lesions (Fig. 3).

All patients of the three groups Group 1 (diode laser), Group II (CO2 laser) Group III (cryosurgery) were assessed for bleeding and pain on the day of the surgery. Evaluation for pain was done with the help of a Visual analog scale (with the extremes having values 0 and 10, with 0 corresponding to ‘no pain’ and 10 corresponding to ‘maximum pain imaginable’). Bleeding was assessed by noting the presence or absence of spontaneous bleed. Edema was evaluated by comparing the wound area with the anatomical area of the opposite side for presence or absence of asymmetry while infection, scar formation and slough were evaluated based on inspection alone.

The follow-up visits were in the first and second postoperative week and pain, edema, slough formation and infection were assessed. Scar formation and lesion recurrence was evaluated at three and six months postoperatively. No intra and postoperative complications or infections were noted subsequently.

-Statistical Analysis

The frequency distribution was expressed in terms of number and percentage for categorical variables (each study parameter) to be compared among three groups. Chi-Square test was used to compare the distribution / association of the study variables between the three groups at each time interval. The mean and standard deviation (SD) was obtained for the pain scores and was compared between the groups using one-way ANOVA test followed by Tukey’s HSD test as the Post hoc analysis. The level of significance (P-Value) was set at *P*<0.05.

All participants have read and signed informed consent form. The use of human subjects in this study has been reviewed and approved by the Dayananda Sagar Institution ethical committee Review Board.

## Results

Pain in the three study groups was compared on the day of surgery, first and second week postoperatively using ANOVA test. On the day of the surgery, the pain was more in the cryosurgery group (mean-2.3±0.5) which was statistically significant when compared with the other two groups.

When the pain score was further compared among CO2 laser group and cryosurgery group, on the day of the surgery, a statistica-lly significant P-value of 0.03 was obtained. In the second week, post-operative comparison of pain score among the three groups showed a significant statistical score (*P*-value of <0.001). Cryosurgery group showed a higher pain score (1.2 ± 0.6) whereas diode laser group showed 100% absence of pain ( Table 1).

**Table 1:**
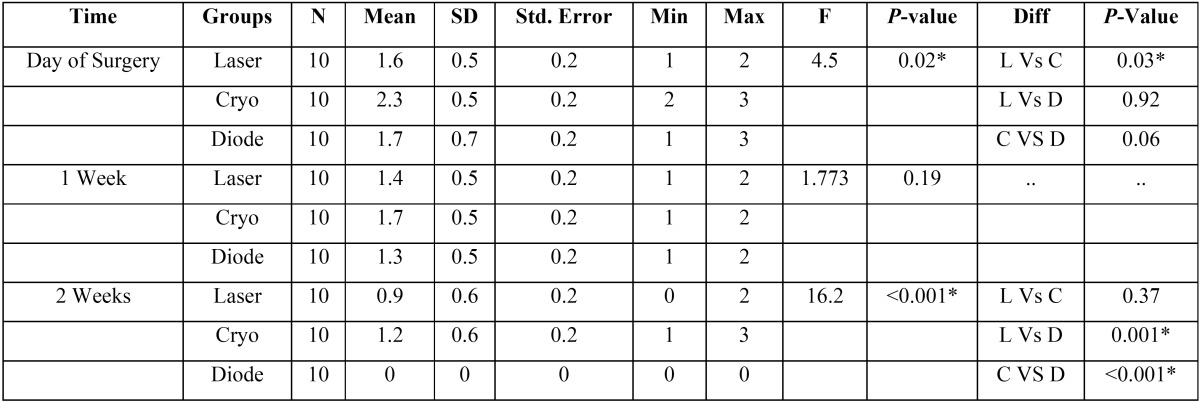
Comparison among the three study groups during Post-op periods using Chi square test.

The presence of edema among the three groups in the first and second postoperative week was analyzed using chi-square test. Edema was recorded in 40% of the participants of cryosurgery group in the first week, whereas in CO2 and Diode laser surgery groups no edema was recorded. A statistically significant *P*- the value of 0.01 was elicited for the first week. However, in the second week no edema was seen in all three groups ( Table 2).

**Table 2:**
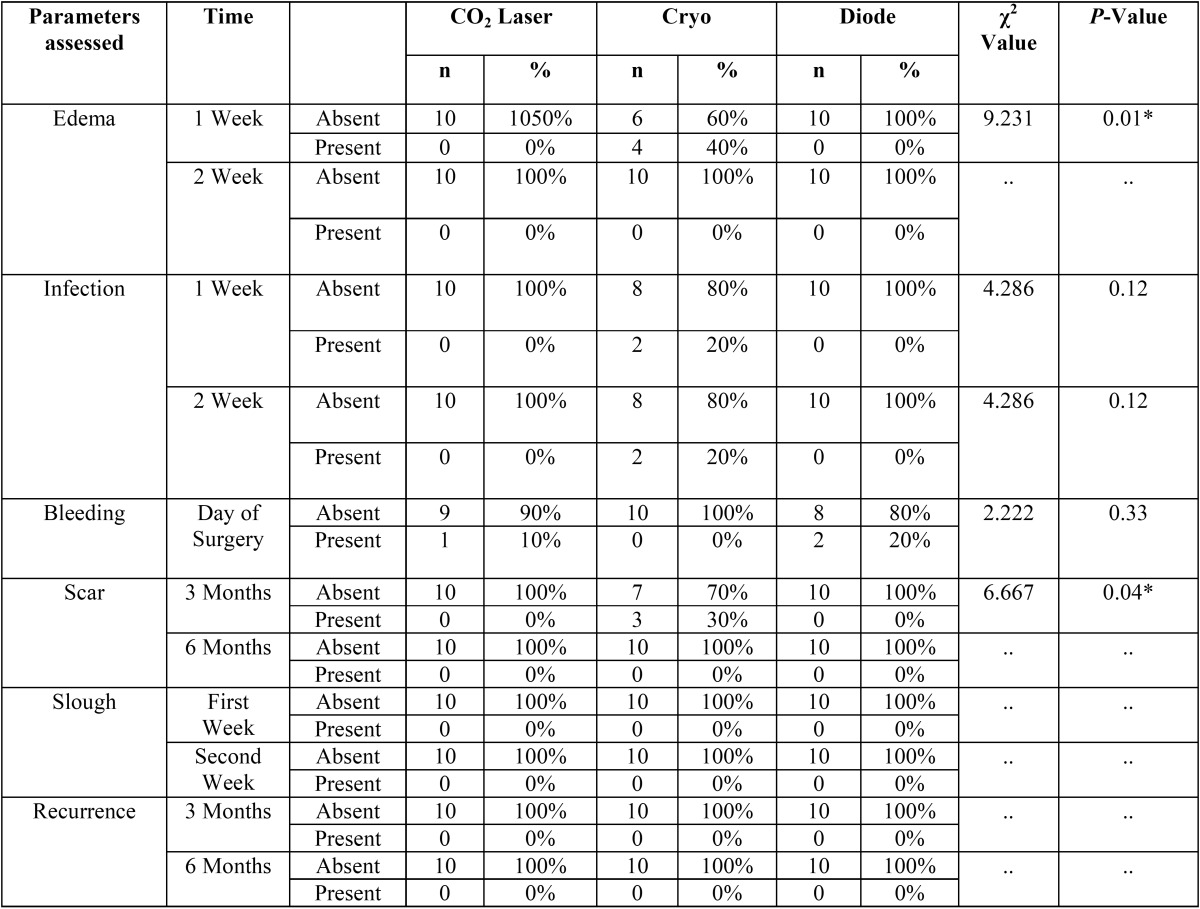
Comparison of Mean Pain score between 03 groups using One-way ANOVA followed by Tukey’s Post hoc Analysis.

Scar formation was observed in 30% of the participants in the Cryosurgery group whereas there was no scar formation in both CO2 laser and Diode laser groups, in the third postoperative month. A statistically significant *P*-value of 0.04 was thus recorded. In the sixth month, the absence of scar formation among all the three groups was noted ( Table 2).

## Discussion

It is an accepted fact that it is indeed a challenge to the clinician to treat OL. It is a potentially pre-malignant lesion with high recurrence capacity and different methods both medical and surgical are employed for its management. Clinical studies done with medical line of management to treat OL have reported high relapse rate with variable side effect severity (14). However, though many clinical studies have been done to evaluate the efficacy of surgical management of OL there are no studies reported comparing the three modalities like cryotherapy, Diode laser and CO2 laser surgery for its effectiveness in treatment of OL. Ideally, any treatment mode must be aimed at being safe, effective minimally invasive, with reduced recurrence or relapse.

Different surgical options have been successfully used for the management of OL lesions which include cryotherapy, diode laser, or CO2 laser (8,11,15). Sako *et al.* have reported complete regression of the lesions using cryotherapy on sixty patients with OL (16). Chin-Jyh Yeh performed cryosurgical treatment on 102 benign oral lesions in which there was no intra operative, or posto-perative bleeding, no surgical defects, minimum scarring, and no infection following treatment (17). Cryotherapy is estimated by authors as a simple, safe, easy, and acceptable treatment modality. Another optimal and safe treatment option in the surgical management of OL is laser surgery. Ben-Bassat *et al.*, first used laser surgery for treatment of OL in 1978 (18). Two types of lasers are used for surgical excision namely diode laser and CO2 laser. Diode laser surgery is well accepted by patients and is an effective mode of treatment due to its bio-stimulating effect which brings about excellent healing and low morbidity (19). The CO2 laser allows vaporization and excision as well as restitution of diseased tissue and provides satisfactory results with low complication rates (20).

In our study, a comparative assessment of cryosurgery, diode laser and CO2 laser surgery in the treatment of OL was performed. All the three modalities can be performed with relative ease and comfort for the patient with minimal use of local anesthesia. As per our study. CO2 and diode laser exhibited significantly better clinical parameters when compared with cryosurgery in the management of leukoplakia. The parameters that were used for evaluating patients were pain, edema and slough formation. This finding is in accordance with clinical studies recorded in literature (21-24) which states that minimal pain in a post- operative period of laser surgery is due to its effect on nerve endings; reduced the thermal damage to surrounding tissue, and sealing of small lymphatic vessels leading to reduced postoperative pain and edema.

Goharkhay *et al.* in their study showed that the diode laser is a very effective because of its excellent coagulation ability (24). In our study, there was a total absence of bleeding in cryosurgery group while CO2 and diode laser surgery groups showed clinically negligible bleeding tendencies. Our observation showed that less thermal damage to the tissues adjacent to the site of injury following the use of diode and CO2 laser. Wound healing after laser surgery at all times was slightly accelerated compared to cryosurgery. Although sloughing was observed on the second day after cryosurgery it was absent when reviewed on the first-week pos-toperatively. Slough was observed in the first few days that could act as a physical barrier causing delayed wound healing. Overall healing followed a comparable time course as there did not appear to be much difference in the rate of repair at the end of the second week. Laser surgery resulted in better wound healing, less contraction, and scarring due to the reduction in the number of myofibroblasts, even in cases of large area of ablation (25).

Scar formation which was seen in patients treated with cryosurgery was totally absent in laser surgery treated groups. However, this scar (following Cryosurgery) settled and disappeared at the end of 6 months postoperatively.

The observations of our study indicated that even though all three surgical modalities used in this study were effective for treatment of leukoplakia, CO2 and diode lasers proved to be a better option in terms of reduced pain intensity, infection, and scar formation. In terms of pain; diode laser proved to be more effective than CO2 laser treatment. After a follow-up of 6 months in all the three study groups, no recurrence was found. The longest follow-up was after 24 months in cryosurgery group, 18 months for CO2 laser group and 12 months for diode laser group. Although the ability of surgical management has not been assessed using randomized clinical trials, in its role in the prevention of recurrent OL and subsequent malignant transformation, this paper has to an extent addressed quantitatively what was achieved by surgical treatment and its prognosis.

## Conclusions

We conclude that the overall summation of the results of the study showed that laser therapy (CO2 and Diode) offered better clinically significant results than cryotherapy in the management of OL. However, as the study group was small and follow-up period was limited, long-term, multicentre randomized, controlled clinical trials are needed to substantiate the efficacy of different surgical methods for treating OL, with emphasis on prevention of recurrence and malignant transformation.
